# Uncovering the clinicopathological features of early recurrence after surgical resection of pancreatic cancer

**DOI:** 10.1038/s41598-024-52909-4

**Published:** 2024-02-05

**Authors:** Hye Yeon Chon, Hee Seung Lee, You-Na Sung, Yoo Keung Tae, Chan Hee Park, Galam Leem, So Jung Kim, Jung Hyun Jo, Moon Jae Chung, Jeong Youp Park, Seung Woo Park, Seung-Mo Hong, Seungmin Bang

**Affiliations:** 1 Division of Gastroenterology, Department of Internal Medicine, Yonsei University College of Medicine, 50-1 Yonsei-ro, Seodaemun-gu, Seoul, 03722 South Korea; 2 Institute of Gastroenterology, Yonsei University College of Medicine, Seoul, South Korea; 3 Department of Pathology, Korea University Anam Hospital, Korea University College of Medicine, Seoul, South Korea; 4 Division of Gastroenterology and Hepatology, Department of Internal Medicine, Ewha Womans University College of Medicine, Seoul Hospital, Seoul, South Korea; 5 Department of Pathology, Asan Medical Center, University of Ulsan College of Medicine, Seoul, South Korea

**Keywords:** Biomarkers, Gastroenterology

## Abstract

To identify risk factors and biomarker for early recurrence in patients diagnosed with pancreatic cancer who undergo curative resection. Early recurrence after curative resection of pancreatic cancer is an obstacle to long-term survival. We retrospectively reviewed 162 patients diagnosed with pancreatic cancer who underwent curative resection. Early recurrence was defined as recurrence within 12 months of surgery. We selected *S100A2* as a biomarker and investigated its expression using immunohistochemistry. Of the total, 79.6% (n = 129) of patients received adjuvant chemotherapy after surgery and 117 (72.2%) experienced recurrence, of which 73 (45.1%) experience early recurrence. In multivariate analysis, age < 60 years, presence of lymph node metastasis, and no adjuvant chemotherapy were significantly associated with early recurrence (all *P* < 0.05). The proportion of patients with high S100A2 expression (H-score > 5) was significantly lower in the early recurrence group (41.5% *vs*. 63.3%, *P* = 0.020). The cumulative incidence rate of early recurrence was higher in patients with an S100A2 H-score < 5 (41.5% vs. 63.3%, *P* = 0.012). The median overall survival of patients with higher S100A2 expression was longer than those with lower S100A2 expression (median 30.1 months *vs*. 24.2 months,* P* = 0.003). High-risk factors for early recurrence after surgery for pancreatic cancer include young age, lymph node metastasis, and no adjuvant therapy. Neoadjuvant treatment or intensive adjuvant therapy after surgery may improve the prognosis of patients with high-risk signatures. In patients who receive adjuvant therapy, high S100A2 expression is a good predictor.

## Introduction

Pancreatic cancer is one of the most disastrous diseases, with a 5-year survival rate of < 5%^1,2^. It is the seventh leading cause of cancer death, increasing by 0.5% to 1.0% per year, and is expected to become the second leading cause of cancer-related mortality by 2030^2,3^. Although late diagnosis of pancreatic cancer with an unresectable status is one of the main reasons, a remarkably high rate of post-operative recurrence is also a major problem^2^. Furthermore, many patients experience early relapse after surgery, raising questions about the role of surgical treatment in pancreatic cancer. Therefore, it is important to identify patients at high risk of early relapse.

Several clinicopathologic risk factors of post-operative recurrence have been suggested^4,5^. These factors include postoperative chemotherapy, increased tumor size, lymph node metastasis, serum carbohydrate antigen (CA 19-9) level, pathological grade, duration of symptoms, and pre-operative modified Glasgow Prognostic Score (mGPS)^6^. However, to date, studies have not provided firm evidence as to which characteristics of patients who can undergo surgery are associated with a high risk of early recurrence^7^.

Recently, the molecular classification of pancreatic cancer has become possible through genome analysis, which helps predict the prognosis of pancreatic cancer more accurately^8^. For example, the squamous subtype according to Moffit’s classification is associated with a poor prognosis compared with the classical subtype. In particular, *TP63* and *S100 calcium binding protein A2* (*S100A2)* have been reported as related genes in the squamous subtype. The S100 family comprises a group of calcium-binding proteins, some of which are important for the development of certain cancer types^9^. However, the biological role of S100A2 protein in pancreatic cancer remains unclear. It has been suggested that it acts as a tumor suppressor in some cases and a promoter in others^10,11^. Also, in several studies, *S100A2* has been reported to correlate with the effects of postoperative chemotherapy^12,13^. However, there have been limited studies regarding biomarkers of early recurrence of pancreatic cancer.

We aimed to identify biomarkers, based on previous transcriptomic analyses, and clinical risk factors for pancreatic cancer associated with early recurrence after surgery.

## Methods

### Study population

**Figure 1:**
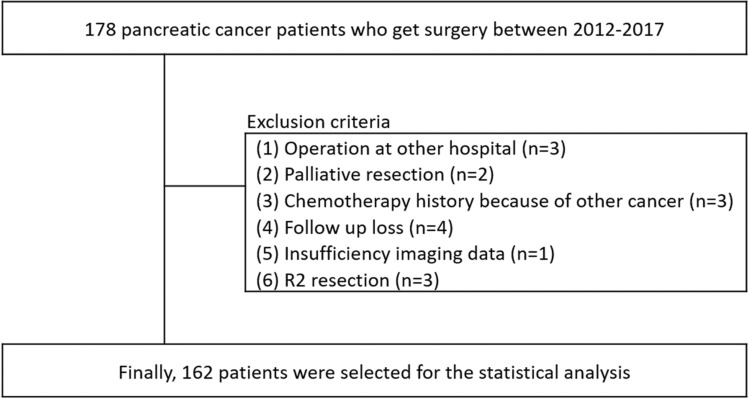
Selection of study population.

This retrospective study was conducted by analyzing patients’ clinical information from Severance Hospital Pancreatic Cancer Cohort Registry database containing tumor information such as tumor grade, operation name, pathological stage and results, and cancer location. The selection of the study population selection is shown in Fig. 1. First, we retrospectively reviewed the medical records of patients diagnosed with pancreatic cancer between September 1, 2012 and February 28, 2017. Cancer diagnoses were classified according to the International Classification of Diseases, 10th Revision (ICD-10) codes. The eligibility criteria were as follows: (1) age > 20 years and < 90 years at diagnosis; (2) underwent surgery for resectable pancreatic cancer; and (3) results of pathology were ductal adenocarcinoma, intraductal papillary mucinous neoplasm with invasive ductal adenocarcinoma, and malignancy (carcinoma). The number of eligible patients was 178 and 16 patients were excluded for the following reasons: (1) underwent surgery at another hospital, hence the specimen of cancer was unavailable (n = 3); (2) underwent palliative resection because of liver metastasis at operation field (n = 2); (3) past history of other cancer and chemotherapy (n = 3); (4) loss at follow up, hence no information about recurrence (n = 4); (5) insufficient imaging data (n = 1); (6) underwent R2 resection (n = 3). Finally, total 162 patients were enrolled to statistical analysis (Fig. 1).

This study conformed to the ethical guidelines of the Declaration of Helsinki (1975) and the need for informed consent was waived by the Independent Institutional Review Board of Severance Hospital (approval no. 4-2015-1058 and 4-2015-0297). Our study has been reported in line with the STROCSS criteria^14^.

### Clinical evaluation and follow-up

Information regarding patient demographics and clinical data were obtained from electronic medical records, including age at diagnosis, sex, location of cancer, pathology, serum level of carbohydrate antigen (CA) 19-9, albumin, white blood cell (WBC) count, lymphocyte count, total bilirubin, antitumor treatment, tumor stage and histologic grade. Mixed location refers to cancer that spans from the head to the body or from the body to the tail of the pancreas. The tumor stage was based on the staging classification of the 7th edition of the American Joint Committee on Cancer (AJCC)^15^. The regimens of adjuvant chemotherapy regimens were based on gemcitabine (n = 103) or 5-fluorouracil (5-FU) (n = 26). During the follow-up period at 3–6 months intervals, patients visited the clinic and underwent abdominopelvic computed tomography (CT) scanning and laboratory tests, including tumor markers.

### Definition of early recurrence

The recurrence interval of 6–12 months after surgical resection for pancreatic cancer has been used as an early recurrence period in previous studies^16–20^. Also, previous studies have reported that the optimal cut-off for differentiating early and late recurrence based on overall survival (OS) was 12 months^4,17^. In this study, we defined early recurrence as recurrence within 12 months after surgery. OS was measured from the date of diagnosis to death from any cause. Tumor recurrence was divided into local and distant tumors.

### Immunohistochemistry

We used resection sample to IHC. We conducted immunohistochemistry of *S100A2* which has been suggested as a biomarker of adjuvant therapy benefits in pancreatic cancer and a marker of the basal-like pancreatic ductal adenocarcinoma subtype^7,12^.

**Figure 2:**
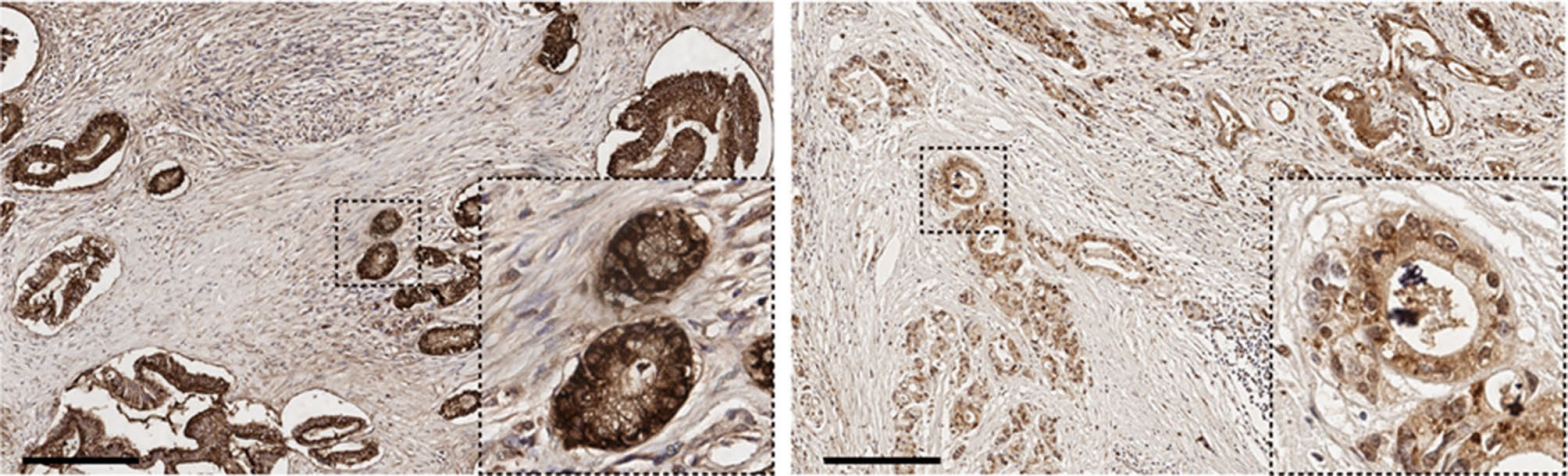
Immunohistochemistry images of S100A2. Left, high S100A2 expression. Right, low to moderate S100A2 expression. Scale bar, 200 µm.

Four-micrometer sections were cut from formalin-fixed paraffin-embedded tissue blocks, deparaffinized, hydrated in xylene, and serially diluted in ethanol. Endogenous peroxidase activity was blocked by incubation with 3% H_2_O_2_ for 10 min. Antigen retrieval was performed with prewarmed 10 mM sodium citrate buffered distilled water (pH 6.0) for 20 min at a 97 °C. The following primary antibodies were used. Primary S100A2 antibodies (mouse monoclonal, catalog no. s6797, 1:200 dilution) were incubated for 18 h at 4 °C. The Dako REAL Peroxidase Detection System Kit was used according to the manufacturer's protocol which included the ready-to-use anti-rabbit/mouse secondary antibody (catalog no. K5007), and counterstained with hematoxylin (catalog no. 03971). All stained slides were evaluated by two experienced board-certified pathologists. Independent two pathologists who blinded to patients’ outcome scored the IHC slides and agreement was accomplished through discussion when the scoring between them was different. A barely discernable light brown nuclear staining of S100A2 only visible at high magnifications (at least 100) was considered "weak," a heterogeneous nuclear staining of varying shades of dark brown "moderate," and a homogeneous dark brown staining "strong" (Fig. 2). The histoscore (H-score) was calculated by multiplying the intensity of staining (graded as 0, negative; 1, weak; 2, moderate; and 3, strong) by the proportion of positive cells from the IHC image^21,22^.

### Statistical analysis

Data were expressed as a mean with standard deviation or median with interquartile range or n (%), as appropriate. One-way ANOVA and chi-squared tests were used to compare the baseline characteristics of patients who were divided into three groups as follows: early recurrence, late recurrence, and no recurrence. Univariate and subsequent multivariate time-dependent Cox proportional hazard models were used to identify the risk factors for early recurrence. Variables which showed statistical significance by *P* < 0.05 in univariate analysis were included to subsequent multivariate analysis. Hazard ratios (HRs) and corresponding 95% confidence intervals (CIs) were calculated. The cumulative incidence rates of early recurrence according to the factors were calculated using the Kaplan–Meier method, and the factors were compared using the log-rank test. The median OS according to these factors was also estimated using the Kaplan–Meier method. A *P* < 0.05 by two-tailed test was considered to indicate significance. All statistical analyses were performed using IBM SPSS statistics for Windows (IBM Corp., Armonk, NY, USA).

### Informed consent waiver

Written informed consent was no required because of the retrospective nature of the study.

This study conformed to the ethical guidelines of the Declaration of Helsinki (1975) and was approved by the Independent Institutional Review Board (approval no. 4-2015-1058) of Severance Hospital, Yonsei University College of Medicine, Seoul, Republic of Korea.

## Results

### Baseline characteristics

The baseline characteristics of the study population are summarized in Table 1. Of the patients, 75.9% were aged > 60 years, and 56.2% were male. Almost patients’ EGOC performance status was 0 (n = 141, 87%) or 1 (n = 20, 12.4%). The tumor location was divided into four sites (head, body, tail, and mixed), and the proportions of the locations were 53.1%, 27.2%, 13.6%, and 6.2%, respectively. The surgical method was selected based on tumor location. When cancer location includes the head of the pancreas, we performed a PPPD. Out of the 10 mixed location, 4 underwent PPPD, while 6 underwent distal pancreatectomy. 77.8% (n = 126) of patients were resectable at baseline. A total of 88.9% (n = 144) of patients underwent R0 resection, and 79.6% (n = 129) received adjuvant chemotherapy after surgery. Histopathological differentiation was evaluated in 144 patients and the result as follows: well (n = 15, 9.3%), moderate (n = 110, 67.9%), poor (n = 19, 11.7%). 17.9% (n = 29) patients were received neoadjuvant therapy. Regarding first-line chemotherapy, 103 (63.6%) patients received gemcitabine-based chemotherapy, and 26 (16.0%) patients received 5-FU based chemotherapy (n = 21, 13.0%) or concurrent chemoradiotherapy (n = 5, 3.0%). The median pre-operative CA 19-9 level was 49.0 U/mL (15.8—239.0 U/mL) which decreased to 30.4 U/mL (9.6–103.7 U/mL) post operation. Also, baseline characteristics of patients with recurrence including epidemiologic features were summarized in Supplementary Table 1. Most of the patients’ ECOG was 0 (n = 98, 83.8%) or 1 (n = 18, 15.4%) and 7 (6.0%) patients had family history of pancareatic cancer. 49 (41.9%) patients had hypertension and 46 (39.3%) patients were diabetes mellitus at diagnosis of pancreatic cancer.

**Table 1:** Baseline characteristics (n = 162).

Variables	
Age, > 60 years	123 (75.9)
Sex	
Male	91 (56.2)
ECOG performance status	
0/1/2	141 (87.0)/20 (12.4)/1 (0.6)
Location of cancer	
Head	86 (53.1)
Body	44 (27.2)
Tail	22 (13.6)
Mixed	10 (6.2)
Tumor size (mm)	30.1 ± 15.3
Resectability	
Resectable/borderline/locally advanced	126 (77.8)/28 (17.3)/8 (4.9)
Surgery	
Pancreaticoduodenectomy	85 (52.5)
Distal pancreatectomy	69 (42.6)
Total pancreatectomy	8 (4.9)
Histological differentiation	
Well	15 (9.3)
Moderate	110 (67.9)
Poor	19 (11.7)
Not evaluated	18 (11.1)
Lymph node status	
N0	87 (53.7)
N1	75 (46.3)
Resection status	
R0	144 (88.9)
R1	18 (11.1)
Neoadjuvant therapy	
Yes	29 (17.9)
No	133 (82.1)
Adjuvant therapy	
Yes	129 (79.6)
No	33 (20.4)
Laboratory variables	
Pre-operative CA 19-9, U/mL	49.0 (15.8, 239.0)
Post-operative CA 19-9, U/mL	30.4 (9.6, 103.7)
Pre-operative albumin, g/dL	3.8 ± 0.5
Pre-operative white blood cell count, 10^3^/µL	6.2 ± 1.9
Pre-operative lymphocyte count, 10^3^/µL	1.8 ± 0.8
Pre-operative total bilirubin, mg/dL	1.2 ± 1.1
S100A2 H-score, ≥ 5 (n = 113)	60 (53.1)

Variables are expressed as mean ± SD or median (IQR) or n (%).

### Recurrence pattern and prognosis

Among the 162 patients, 117 (72.2%) experienced recurrence, of which 73 (45.1%) experienced early recurrence. The locations of recurrence sites are shown in Supplementary Table 2. Disease recurrence was classified as follows: (1) local recurrence (in the pancreatic resection bed, mesentery, regional lymph node or soft tissue around the SMA, SMV, and celiac axis) and (2) distant recurrence (hepatic, pulmonary, peritoneum, and other distant organs). The most common sites of local and distant recurrence were the regional lymph nodes or soft tissue around the SMA, SMV (n = 16, 59.3%), and liver (n = 43, 47.8%). The recurrence pattern in patients with early recurrence is depicted in Supplementary Fig. 1 according to the treatment strategy. Among the 73 patients who developed early recurrence, 15 (20.5%) experienced local recurrence and 58 (79.4%) developed distant recurrence. The recurrence pattern did not differ according to the treatment strategy.

### Comparison between patients with and without early recurrence

We compared the patients with early recurrence to those without early recurrence (late recurrence and no recurrence; Table 2). The proportion of patients aged > 60 years was higher in the no recurrence group than in the early recurrence and late recurrence groups. (65.8% *vs*. 84.1% *vs*. 84.4%, *P* = 0.023). Proportion of resectable patients were higher in no recurrence group (n = 41, 91.1%) than early recurrence (n = 50, 68.5%) and late recurrence (n = 35, 79.5%) group with marginal significance (*P* = 0.052). Also, the proportion of patients with N1 stage was higher in early recurrence group (72.6% *vs*. 29.5% *vs*. 20.0 *P* < 0.001). Preoperative albumin level was lower in patients with early recurrence (3.7 g/dL *vs*. 3.9 g/dL *vs*. 4.0 g/dL, *P* = 0.008), whereas more patients presented with higher CA19-9 (> 49 U/mL) in the early recurrence group (56.3% *vs*. 52.3% *vs*. 33.3%, *P* = 0.047). The proportion of patients with an S100A2 H-score > 5 was lower in the early recurrence group (41.5% *vs*. 58.6% *vs*. 67.7, *P* = 0.053).

**Table 2:** Comparison characteristics of patients with and without early recurrence.

Variables	Early recurrence	Late recurrence	No recurrence	*P* value
	(n = 73, 45.1%)	(n = 44, 27.2)	(n = 45, 27.8)	
Age, > 60 years	48 (65.8)	37 (84.1)	38 (84.4)	0.023
Sex (male)	40 (54.8)	20 (45.5)	31 (68.9)	0.079
ECOG performance status				
0/1/2	60 (82.2)/12 (16.4)/1 (1.4)	38 (86.4)/6 (13.6)/0	43 (95.6)/2 (4.4)/0	0.275
Location of cancer				0.302
Head	32 (43.8)	29 (65.9)	25 (55.6)	
Body	25 (34.2)	9 (20.5)	10 (22.2)	
Tail	10 (13.7)	4 (9.1)	8 (17.8)	
Mixed	6 (8.2)	2 (4.5)	2 (4.4)	
Tumor size (mm)	30.1 ± 13.5	29.2 ± 11.9	31.1 ± 20.4	0.810
Resectability				
Resectable/borderline/locally advanced	50 (68.5)/19 (26.0)/4 (5.5)	35 (79.5)/6 (13.6)/3 (6.8)	41 (91.1)/3 (6.7)/1 (2.2)	0.052
Surgery				0.109
Pancreaticoduodenectomy	46 (63.0)	23 (52.3)	15 (33.3)	
Distal pancreatectomy	25 (34.2)	19 (43.2)	25 (55.6)	
Total pancreatectomy	2 (2.7)	2 (4.5)	4 (8.9)	
Histological differentiation				0.141
Well	5 (6.8)	3 (6.8)	7 (15.6)	
Moderate	50 (68.5)	31 (70.5)	29 (64.4)	
Poor	13 (17.8)	4 (9.1)	2 (4.4)	
Lymph node metastasis				< 0.001
No	20 (27.4)	31 (70.5)	36 (80.0)	
Yes	53 (72.6)	13 (29.5)	9 (20.0)	
Resection margin				0.203
R0	62 (84.9)	39 (88.6)	43 (95.6)	
R1	11 (15.1)	5 (11.4)	2 (4.4)	
Neoadjuvant therapy				0.998
Yes	13 (17.8)	8 (18.2)	8 (17.8)	
No	60 (82.2)	36 (81.8)	37 (82.2)	
Adjuvant therapy				0.129
Yes	53 (72.6)	38 (86.4)	38 (84.4)	
No	20 (27.4)	6 (13.6)	7 (15.6)	
Laboratory variables				
Pre-operative CA 19-9, ≥ 49 U/mL	40 (56.3)	23 (52.3)	15 (33.3)	0.047
Post-operative CA 19-9, ≥ 30 U/mL	43 (61.4)	20 (45.5)	17 (37.8)	0.054
Pre-operative albumin, g/dL	3.7 ± 0.6	3.9 ± 0.4	4.0 ± 0.5	0.008
Pre-operative white blood cell count, 10^3^/µL	6.4 ± 2.1	6.0 ± 1.7	6.2 ± 1.7	0.711
Pre-operative lymphocyte count, 10^3^/µL	1.7 ± 0.7	1.8 ± 0.7	1.8 ± 0.8	0.375
Pre-operative total bilirubin, mg/dL	1.35 ± 1.28	1.00 ± 0.80	0.98 ± 0.87	0.549
S100A2 score, ≥ 5 (n = 113)	22 (41.5)	17 (58.6)	21 (67.7)	0.053

Variables are expressed as mean ± SD or n (%).

### Risk factors of early recurrence

We conducted a Cox analysis to determine the risk factors for early recurrence after curative resection compared with the late or no recurrence groups (Table 3). In the univariate analysis, age < 60 years, presence of lymph node metastasis, borderline or locally advanced resectability, no adjuvant chemotherapy, post-operative CA 19-9 level > 30 U/mL, decreased pre-operative albumin level, increased pre-operative total bilirubin level, and S100A2 H-score < 5 were significantly associated with early recurrence. Age < 60 years (HR = 0.416, 95%CI = 0.217–0.795), presence of lymph node metastasis (HR = 4.033, 95% CI = 2.091–7.781), and no adjuvant chemotherapy (HR = 2.400, 95% CI = 1.198–4.807) were independent risk factors for early recurrence in multivariate analysis (All *P* < 0.05).

**Table 3:** Risk factors associated with early recurrence in patients with curative resection.

Variables	Univariate			Multivariate		
	HR	95% CI	*P* value	HR	95% CI	*P* value
Age, > 60 years	0.508	0.312–0.826	0.006	0.423	0.220–0.813	0.010
Lymph node metastasis						
Yes (*vs*. no)	4.760	2.831–8.005	< 0.001	3.949	2.038–7.655	< 0.001
Resectability						
Resectable (*vs*. borderline or locally advanced)	0.482	0.293–0.791	0.004	0.858	0.431–1.711	0.664
Adjuvant therapy						
No (*vs*. yes)	2.550	1.521–4.275	< 0.001	2.269	1.083–4.757	0.030
Laboratory variables						
Pre-operative CA 19-9, ≥ 49 U/mL	1.524	0.953–2.437	0.078	–	–	–
Post-operative CA 19-9, ≥ 30 U/mL	1.728	1.068–2.798	0.026	1.544	0.798–2.989	0.197
Pre-operative albumin, g/dL	0.469	0.294–0.747	0.001	0.790	0.439–1.420	0.430
Pre-operative total bilirubin, mg/dL	1.281	1.054–1.558	0.013	0.895	0.671–1.195	0.452
S100A2 H-score, ≥ 5 (n = 113)	0.503	0.291–0.870	0.014	0.737	0.413–1.318	0.304

HR, hazard ratio; CI, confidence interval.

### S100A2 as a biomarker for early recurrence

**Figure 3:**
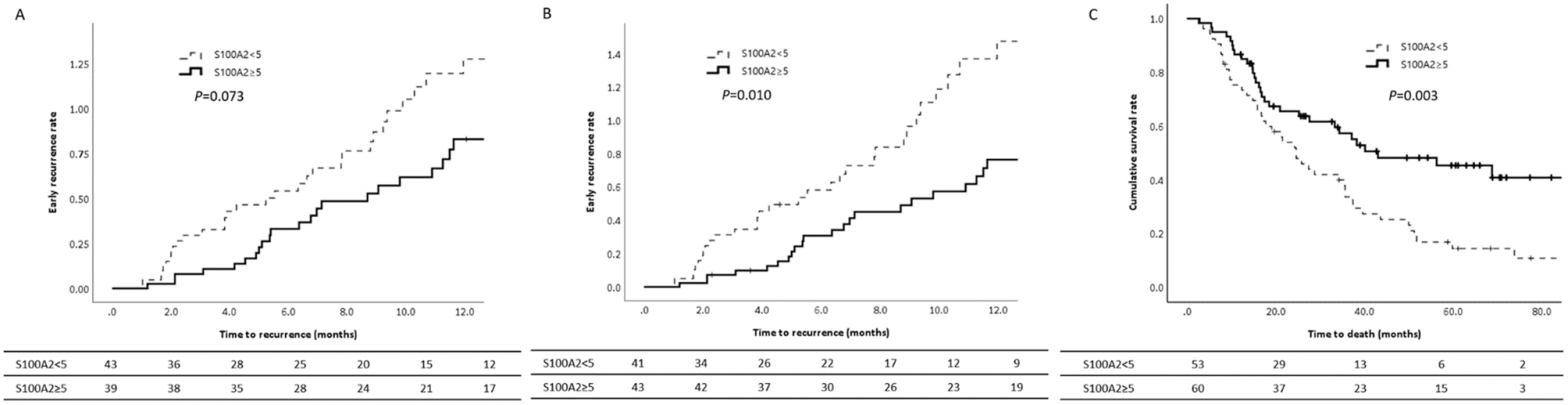
The cumulative early recurrence rate in patients with recurrence (**A**) and without late recurrence (**B**) according to S100A2 H-score. The cumulative survival rate according to S100A2 H-score in total patients (**C**).

Patients were divided into low and high S100A2 expression groups (Fig. 3) followed by a cut-off point 5 of the S100A2 H-score, since this cut-off point significantly discriminated between patients with early recurrence and those without early recurrence (*P* = 0.012; Fig. 3A and 3B). The cumulative incidence rate of early recurrence was higher in patients with S100A2 H-scores < 5. Patients with high S100A2 expression (H-score, ≥ 5) had significantly longer OS than those with low S100A2 expression (median 30.1 months *vs*. 24.2 months, *P* = 0.003; Fig. 3C). The subgroup analysis according to adjuvant therapy is shown in Supplementary Fig. 2. In patients who received adjuvant therapy, patients with high S100A2 expression (H-score, ≥ 5) had significantly longer OS than those with low S100A2 expression (median 33.3 months *vs*. 27.4 months, *P* = 0.011). In contrast, there was no difference in OS between the subgroups who did not receive adjuvant therapy. Also, we conducted subgroup analysis to evaluate impact of neoadjuvant therapy. Among 113 patients with S100A2 score, 19 patients received neoadjuvant therapy. In subgroup analysis in patients without neoadjuvant therapy (n = 94), S100A2 score was significantly associated with early recurrence (HR = 0.412, 95% CI = 0.224–0.758, P = 0.004). However, we could not analysis in the subgroup with neoadjuvant therapy, because the sample size was too small to evaluate the function of S100A2 score.

## Discussion

Although early recurrence after surgery for pancreatic cancer is a clinically important marker of poor prognosis, few studies have described the clinico-pathological characteristics of patients with early recurrence. We used 12 months as the cut-off to diagnose early recurrence after pancreatectomy, according to a previous study^16,17^. Our study implied that younger age, lymph node metastasis, lower pre-operative albumin and higher post-operative bilirubin were significantly associated with early recurrence in patients with curative resection. By contrast, adjuvant chemotherapy was independently associated with a reduced risk of early recurrence. In addition, S100A2 H-score was negatively associated with early recurrence. The cumulative survival rate in patients with an S100A2 H-score of > 5 was higher than that in patients with an S100A2 H-score of < 5.

NCCN guidelines recommend adjuvant chemotherapy after resection to prevent cancer recurrence^23^. Suto et al. have described the influence of post-operative adjuvant therapy^24^. Early distant recurrence was observed in only 9% patients who underwent surgery plus post-operative adjuvant chemotherapy. But, it occurred in 44% who underwent surgery alone (*P* = 0.001). In our cohort, the incidence of early recurrence was lower in patients who underwent adjuvant chemotherapy or chemoradiotherapy (n = 53/129, 41%) than in those who underwent surgery alone (n = 20/33, 61%). In early local recurrence, 11 (73.4%) cases occurred in patients who received adjuvant therapy and four (26.7%) patients who did not receive adjuvant therapy. Despite adjuvant therapy, early distant recurrence (n = 35, 60.3%) occurred as often as early local recurrence (n = 7, 46.7%) in patients who underwent surgery with adjuvant chemotherapy or chemoradiotherapy. Although adjuvant chemotherapy prevents early recurrence, further studies are needed to investigate the impact of adjuvant chemotherapy on recurrence patterns after surgery.

In previous studies, high CA 19-9 level, large tumor size, poor tumor differentiation grade, neutrophil–to–lymphocyte ratio, resection margins, and lymph node metastasis have been associated with an increased likelihood of early recurrence^4,5,17,25,26^. In contrast, adjuvant therapy was associated with a reduced likelihood of early recurrence^4,17,20,25,27^. However, a clear biochemical marker of early recurrence after pancreatic cancer resection is still lacking.

In our study, high S100A2 expression was associated with lower rates of early recurrence and higher OS. Ohuchida et al. have described how S100A2 could be involved in the pathogenesis of pancreatic cancer and that it is correlated with the metastasis of pancreatic cancer^28^. Also, in a study involving a large homogeneous cohort of patients with resected pancreatic cancer, S100A2 expression was strongly associated with better OS and RFS in patients who received adjuvant treatment, whereas no statistical trend was observed in patients treated with surgery alone^12^. This means that S100A2 protein expression could predict the benefit of adjuvant treatment in patients with resected pancreatic cancer (Supplementary Table 3). In our cohort for S100A2 expression analysis cohort, 129 (79.6%) patients received adjuvant chemotherapy or chemoradiotherapy. High S100A2 expression was significantly associated with a longer OS. In contrast, the prognostic value of S100A2 expression was diminished in patients who underwent surgery alone. This might indicate that the survival benefit from adjuvant chemotherapy is higher in patients with high S100A2 expression than in those with lower S100A2 expression through the suppression of the metastatic risk related to S100A2 expression.

In our study, most patients (79.5%) with early recurrence had distant metastasis. The incidence of hepatic metastasis was significantly higher in the early recurrence group than in the late recurrence group (60.5 vs. 39.5%, respectively). Similar results have also been reported in several previous studies^29,30^. Sarang Hong et al. reported 73.5% (72/98) with distant recurrence occurred in early recurrence group compared to 64.6% (133/309) with distant recurrence in late recurrence group. In another study, the incidence of hepatic recurrence was significantly higher in the early recurrence group than in the late recurrence group (39.7 vs. 15.4%, *P* = 0.003). This means that there is a possibility of early micro-metastasis during cancer diagnosis, even if there is no mass on CT. If these patients were subdivided into resectable or borderline resectable groups, we would need to know their characteristics to prevent early recurrence and perform neoadjuvant chemotherapy to control micro-metastasis. The high recurrence rate has been attributed to the presence of occult micro-metastatic disease at the time of resection. Our team have previously reported the predictive risk factors related to occult distant metastasis, which were not detected on radiologic images, and the efficacy of neoadjuvant therapy in resectable pancreatic cancer^31,32^.

Our study had several limitations. First, it was a retrospective study with a small sample size. Therefore, a selection bias may have been present in patient selection. Second, the cohort was a heterogeneous mix of patients with/without adjuvant chemotherapy and neoadjuvant chemotherapy. Third, we could not evaluate ECOG performance status after surgery as a predictor of early recurrence because lack of the data. ECOG performance status after surgery is an important factor of decision of adjuvant therapy, therefore patients who didn’t receive adjuvant therapy due to poor general condition might have experienced more relapse. Fourth, although previous studies reported a similar efficacy of S100A2 in patients who received adjuvant chemotherapy, it has recently been shown that increased S100A2 expression is associated with squamous and basal-type pancreatic cancers with poor prognosis. Previous studies have also reported that the basal type may have gemcitabine responsiveness and a better response to adjuvant therapy^33,34^. In surgery only group, shorter mOS might be expected in patients with high S100A2 score than those with low S100A2, but mOS of those two groups was similar in our cohort. Maybe, the number of patients who underwent only surgery was too small to evaluate the prognostic function of S100A2 in the subgroup of our cohort. Further research is needed to identify the tumor suppressor role of S100A2 expression associated with adjuvant chemotherapy in well-designed studies using large sample size.

In conclusion, we identified clinicopathological factors related to the early recurrence of surgically resected pancreatic cancer. Neoadjuvant treatment has recently been proposed as an alternative treatment for patients in high-risk relapse groups. It may be helpful to select patients with high-risk signatures who need to be treated with neoadjuvant or intensive adjuvant therapy after surgery because of their predicted lower disease-free survival. Furthermore, S100A2 expression can be used to identify individuals who may benefit from adjuvant chemotherapy.

### Supplementary Information

Supplementary Information.

## Data Availability

The data generated or analyzed during this study available from the corresponding author on reasonable request.

## Abbreviations

CA9-9: Carbohydrate antigen
mGPS: Glasgow prognostic score
S100A2: S100 calcium binding protein A2
WBC: White blood cell
AJCC: American Joint Committee on Cancer
CT: Computed tomography
OS: Overall survival
IHC: Immunohistochemistry
H-score: Histoscore
HRs: Hazard ratios
CIs: Confidence intervals
