# Supplementary material for: Uncovering the clinicopathological features of early recurrence after surgical resection of pancreatic cancer
Source: Sci Rep. 2024 Feb 5;14:2942. doi: 10.1038/s41598-024-52909-4 (PMC10844252; doi:10.1038/s41598-024-52909-4)
Supplement: Supplementary file 1 — Supplementary Information. [file 41598_2024_52909_MOESM1_ESM.docx]

**Supplementary Figure 1.** Recurrence pattern in patients with early recurrence


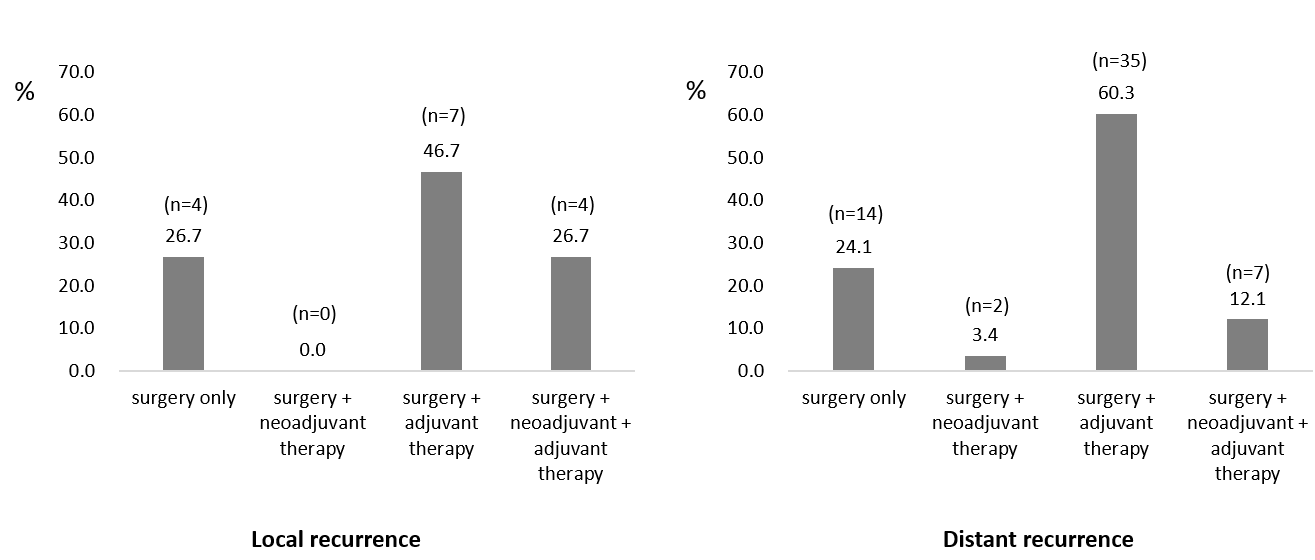


**Supplementary figure 2.** The cumulative early recurrence rate in patients with recurrence, in patients who received adjuvant therapy (A) and in those without adjuvant therapy (B)


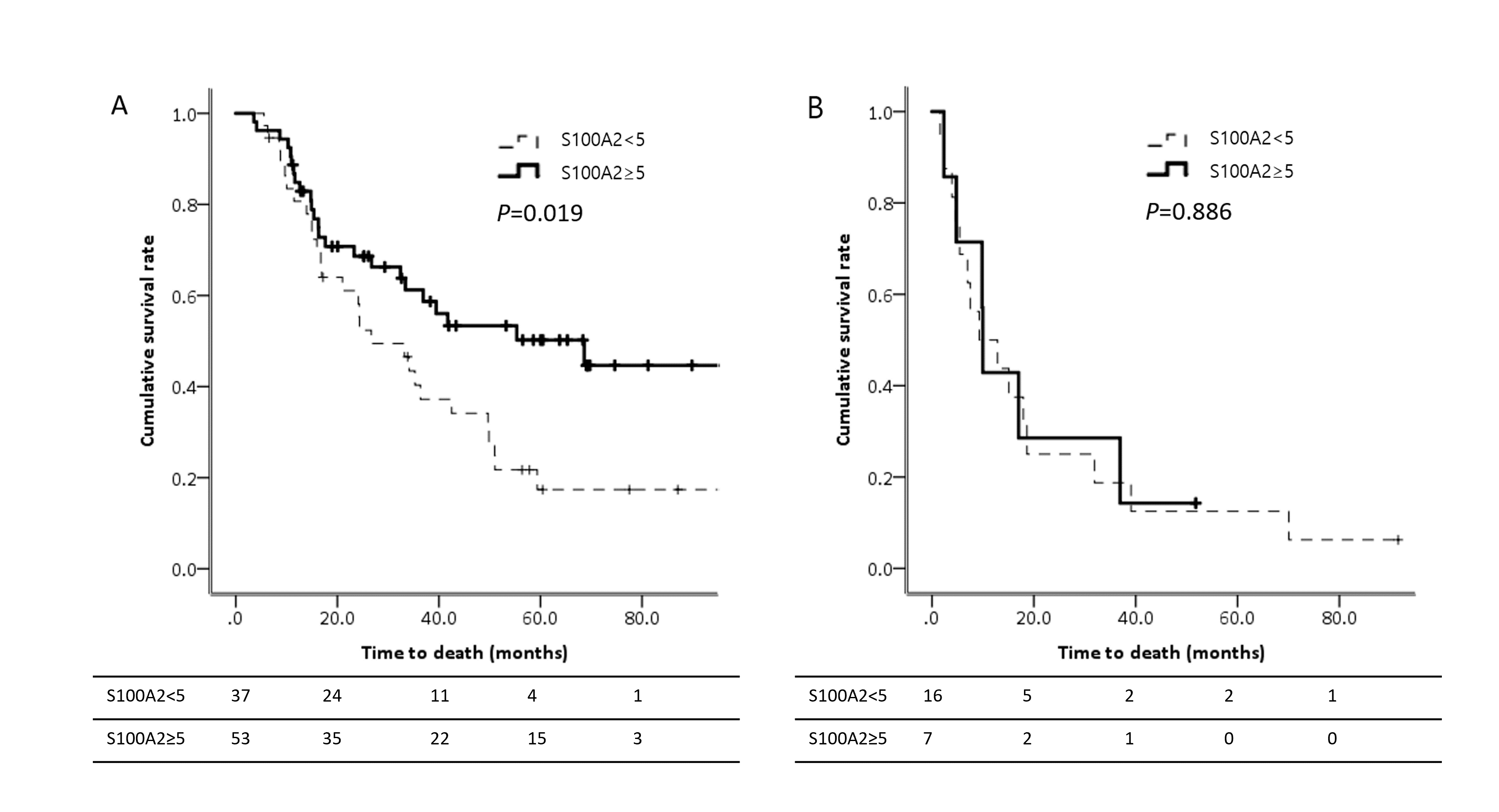


| **Supplementary table 1.** Baseline characteristics of patient with recurrence (n = 117) | |
| --- | --- |
| Variables |  |
| Age, >60 years | 85 (72.6) |
| Sex |  |
| Male | 60 (51.3) |
| ECOG |  |
| 0/1/2 | 98 (83.8)/18 (15.4)/1 (0.9) |
| Body mass index | 23.0 (21.0, 24.7) |
| Family history of pancreatic cancer | 7 (6.0) |
| Hypertension | 49 (41.9) |
| Diabetes mellitus | 46 (39.3) |
| Smoking |  |
| None/Ex/Current | 68 (58.1)/29 (24.8)/20 (17.1) |
| Alcohol consumption |  |
| None/Ex/Current | 64 (54.7)/24(20.5)/29(24.8) |
| Location of cancer |  |
| Head | 61 (52.1) |
| Body | 34 (29.1) |
| Tail | 14 (12.0) |
| Mixed | 8 (6.8) |
| Tumor size (mm) | 29.7 ± 12.9 |
| Resectability |  |
| resectable/borderline/locally advanced | 85 (72.6)/25(21.4)/7(6.0) |
| Surgery |  |
| Pancreaticoduodenectomy | 69 (61.5) |
| Distal pancreatectomy | 44 (37.6) |
| Total pancreatectomy | 1 (0.9) |
| Histological differentiation |  |
| Well | 8 (6.8) |
| Moderate | 81 (69.2) |
| Poor | 17 (14.5) |
| Not evaluated | 11 (9.4) |
| Lymph node status |  |
| N0 | 51 (43.6) |
| N1 | 66 (56.4) |
| Resection status |  |
| R0 | 101 (86.3) |
| R1 | 16 (13.7) |
| Neoadjuvant therapy |  |
| Yes | 21 (17.9) |
| No | 96 (82.1) |
| Adjuvant therapy |  |
| Yes | 91 (77.8) |
| No | 26 (22.2) |
| Laboratory variables |  |
| Pre-operative CA 19-9, U/mL | 83.4 (17.3, 273.0) |
| Post-operative CA 19-9, U/mL | 37.3 (9.2, 125.2) |
| Pre-operative Albumin, g/dL | 3.8 ± 0.5 |
| Pre-operative White blood cell count, 10^3/µL | 6.3 ± 1.9 |
| Pre-operative Lymphocyte count, 10^3/µL | 1.7± 0.7 |
| Pre-operative Total bilirubin, mg/dL | 1.2 ± 1.1 |
| S100A2 H-score, ≥ 5 (n=82) | 39 (47.6) |
| Variables are expressed as mean ± SD or median (IQR) or n (%). | |

| **Supplementary table 2**. Location of recurrence | | |
| --- | --- | --- |
| Recurrence site | | N (%) |
| Local recurrence | Pancreatic resection bed | 10 (37.0) |
|  | Regional lymph node or soft tissue (SMA, SMV) | 16 (59.3) |
|  | Nodular lesion at celiac axis | 1 (3.7) |
| Distant recurrence | Liver | 43 (47.8) |
|  | Lung | 13 (14.4) |
|  | Peritoneum | 27 (30.0) |
|  | Other distant organ | 7 (7.8) |
| Total | | 117 |

**Supplementary table 3.** Previous studies related to the role of S100A2 after resection in pancreatic cancer

|  | Ohuchida, K. et al.  J Pathol. 2007 | Biankin, A. V. et al. Gastroenterology. 2009 | Bachet, J. B. et al.  Eur J Cancer. 2013 | Our study. 2023 |
| --- | --- | --- | --- | --- |
| Sample size | n=24 | n=296 | n=471 | n=162 |
| Patients number who received adjuvant chemotherapy | N/A | 55 (18.8%) | 329 (70%) | 129 (79.6%) |
| Key results | S100A2 expression was higher in samples from patients who survived < 1000 days after surgery than in those from patients who survived > 1000 days. | Patients with S100A2-negative tumors had a significant survival benefit from pancreatectomy | S100A2 expression predicts longer DFS and OS in patients treated with adjuvant therapy and should be evaluated as a predictive biomarker | The cumulative incidence rate of early recurrence was higher in patients with low S100A2 |
